# Donders is dead: cortical traveling waves and the limits of mental chronometry in cognitive neuroscience

**DOI:** 10.1007/s10339-015-0662-4

**Published:** 2015-07-03

**Authors:** David M. Alexander, Chris Trengove, Cees van Leeuwen

**Affiliations:** Brain & Cognition Research Unit, University of Leuven, Leuven, Belgium; Kaiserslautern University of Technology, Kaiserslautern, Germany

**Keywords:** Cortex, Traveling waves, Difference method

## Abstract

An assumption nearly all researchers in cognitive neuroscience tacitly adhere to is that of space–time separability. Historically, it forms the basis of Donders’ difference method, and to date, it underwrites all difference imaging and trial-averaging of cortical activity, including the customary techniques for analyzing fMRI and EEG/MEG data. We describe the assumption and how it licenses common methods in cognitive neuroscience; in particular, we show how it plays out in signal differencing and averaging, and how it misleads us into seeing the brain as a set of static activity sources. In fact, rather than being static, the domains of cortical activity change from moment to moment: Recent research has suggested the importance of traveling waves of activation in the cortex. Traveling waves have been described at a range of different spatial scales in the cortex; they explain a large proportion of the variance in phase measurements of EEG, MEG and ECoG, and are important for understanding cortical function. Critically, traveling waves are not space–time separable. Their prominence suggests that the correct frame of reference for analyzing cortical activity is the dynamical trajectory of the system, rather than the time and space coordinates of measurements. We illustrate what the failure of space–time separability implies for cortical activation, and what consequences this should have for cognitive neuroscience.

**Electronic supplementary material:**

The online version of this article (doi:10.1007/s10339-015-0662-4) contains supplementary material, which is available to authorized users.

## Donders’ method

Franciscus Cornelis Donders (1818–1889) was the first scientist to apply the *subtraction method* to cognition. By comparing simple to choice reaction times, he was able to draw conclusions regarding the time course of choice processing.

The idea occurred to me to interpose into the process of physiological time some new components of mental action. If I investigated how much this would lengthen the physiological time, this would, I judged, reveal the time required for the interposed term

(Donders 1969, p. 418)

This idea is now a central tenet of cognitive neuroscience. The point is that we can take one cognitive process (possibly a baseline or resting state) and compare a second process that differs in only one important respect. The difference between two cognitive processes is revealed in the difference between the two sets of measurements associated with them. Further, the properties of the extra cognitive component can be isolated by making this subtraction. These properties involve the time course of the extra component (in Donders’ example) or brain activation patterns in the case of fMRI, EEG and MEG.

The subtraction method allows cognitive operations to be inferred in a straightforward manner by subtracting measurements from a baseline condition. That the results are thereby meaningful derives from what we shall call the assumption of additivity. The measured signal can be described as a linear combination of different signal sources. This assumption is of primary importance for the subtraction method. However, an additional assumption is often implicit in how differencing is usually undertaken in neuroscience. This assumption is called space–time separability. We will argue that the failure of space–time separability in neuroscience means that the assumption of additivity is misleadingly applied.

As far back as 1868, Donders understood the potential limits and shortcomings of his method:

It is readily seen that the course taken in the research was not irreproachable… If one enters a room from two sides successively to do something there, it is unlikely that in both cases one will leave the room through a third door within the same interval of time… Thus it is not surprising that in repetitions of experiments, mainly keeping to the same method, very divergent results were obtained. (ibidem, pp. 415–416).

Here we will argue that the problem expressed by the ‘different doors’ metaphor is much more common than the widespread use of the difference method in cognitive neuroscience would suggest. The key empirical evidence comes from recent insights into traveling waves of activity in the cortex. We suggest (but will not fully demonstrate) that the problem is an in-principle one. So the problem arises at the base of theory, not simply as a limitation of methods. In this sense, the problem is akin to the errors of the four humors, the four classical elements, or spontaneous generation. It is not akin to flat earth as a local approximation, Newtonian mechanics at low velocities, or methodological conveniences such as ignoring air resistance in the measurement of falling bodies.

## Space–time separability in neuroscience

Cortical signals are measured at different brain locations and over a range of times. The activity measurements may be in microvolts (in the case of EEG) or femtoTesla (in the case of MEG) or rise and fall of Blood-oxygen-level-dependent (BOLD) response (in the case of fMRI) (Boynton et al. 1996). The different spatial locations are determined by the position of an EEG or MEG sensor or the coordinates of an imaged voxel of fMRI. Each measurement type has a characteristic time course, whether it involves oscillations in the alpha-band at the 100-ms timescale, or the rise and fall of brain metabolic measures over a few seconds, in the case of fMRI.

A ubiquitous methodological practice in cognitive neuroscience is to obtain measure of brain activity by analyzing the time course of activity alone, or the spatial topography of activity alone. This usually results in throwing away most of the data as irrelevant: It is considered enough to analyze the time series at a site of interest, or to take spatial snapshots at some relevant times. This practice boils down to treating brain data as if it were space–time separable.

The assumption of space–time separability in cognitive neuroscience is usually left implicit, but covers quite a range of methods. Before continuing on to the evidence as to why this assumption does not hold, we will spell out the implications as follows:

1. The additivity assumption states that cortical activity may be considered as a sum of component patterns of activity.
2. Each component pattern can be represented as (a) a topographic array of activations multiplied by the (b) corresponding task/stimulus-locked activation time series.
3. A consequence of (2) is that (a) and (b) may be considered separately in further analyses as separable functions of space and time, respectively.
4. The separate component functions of the signal, defined by (3), can each be mapped into a common temporal or spatial coordinate system.
5. The mapping in (4) can be achieved by affine linear transformations of either the time domain or the spatial domain, e.g., scaling and translation of the data.
6. The scaling and translation of the spatial or temporal domain are constant for a given subject, task condition, or signal type of interest. This enables averaging the signals within conditions or the function type of interest.

According to additivity assumption (1), the total activity measured during an experiment is assumed to be the sum of task-related component *A*_1_ and other non-task components *A*_2_ … *A*_*n*_. The first component derives from the cognitive function of interest, the others from the baseline task. The difference method states that the baseline condition *A*_2_ … *A*_*n*_ can be subtracted from *A*_1_, *A*_2_ … *A*_*n*_ leaving only the activity of interest, *A*_1_.

**Fig. 1:**
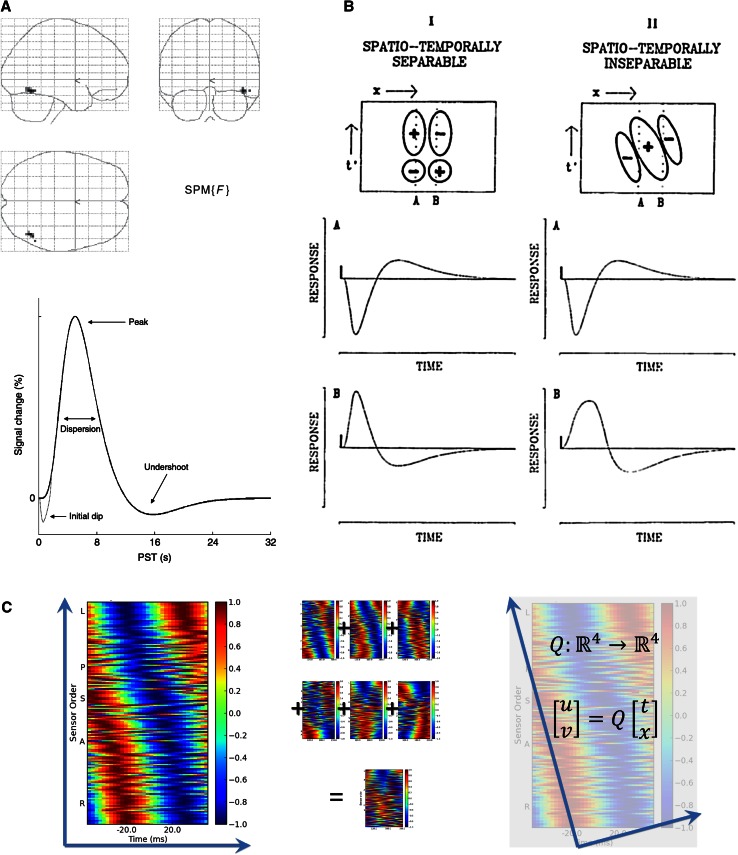
Examples of space–time separability and inseparability in neuroscience. **a** Standard fMRI imaging techniques decompose the BOLD response into a hemodynamic response function and a voxel map of amplitudes. Here we show a map of cortical amplitudes to which a threshold has also been applied, for ease of visualization. Convolving the response function with the unthresholded amplitude map reproduces the original signal, under the assumption that the two are space–time separable. **b** Many orientation-selective receptive fields in the primary visual cortex are space–time separable. This means that the time course of the response differs by only amplitude and sign, and not in shape, depending on the exact spatial location and direction of the visual stimulation. However, many direction-selective cells have receptive fields that are space–time inseparable. This means that the time course of the response will change shape depending on the exact spatial location and direction of the stimulation. **c** MEG traveling wave shown as the cosine of the phase within the time–space plot. The phase of the wave is a function of both space and time, showing the characteristic diagonal symmetry (*left*). However, the axis symmetry changes from trial to trial, washing out most of the signal when trial averages are made (*middle*). A transformation of coordinates is required in order for traveling waves to be additive (*right*)

According to (2), the activity of interest, *A*_1_, is understood as signal measurements in space, *x*, and time, *t,* such that:

$$A_{1} \left( {t,x} \right) = f\left( t \right) \times g\left( x \right),$$

where *f* and *g* are independent. What this means is that one component of *A*_1_ is a function of time, and only time, and one component is a function of space, and only space. Examples of such functions are given in Fig. 1. Panel A (upper) shows a topographical map of cortical activation, typical in fMRI. This map corresponds to *g*(*x*) after thresholding. Two conditions (usually one of them a baseline) are compared by taking a difference map. Panel A (lower) shows the typical time course of the hemodynamic response. This corresponds to *f*(*t*) and is obtained after spatial averaging (6). Then, the time course of the signal is compared to a model signal. The model signal is constructed by convolving expected event signals with the hemodynamic response function. The correlation of the brain signal and the model is done for each location separately (3). If the goal of the fMRI study is the spatial topography of brain events, individual topographies are made to correspond within a common coordinate system (4), averaged within conditions (6) and compared between conditions via the difference method (1). At each step, the signal is considered as either a spatial topography or a time series (3), and alternating between these two views results in maps of localized function familiar in fMRI studies.

**Fig. 2:**
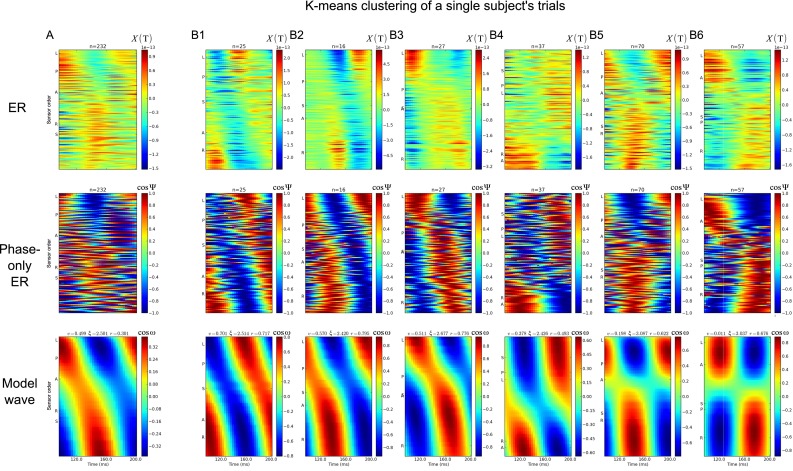
Clustering trials by phase values reveals a wide range of dynamics in sub-averages. These data are taken from a single subject. The phase values over one temporal cycle (108 ms duration for one cycle at 9.2 Hz) are grouped together over all the measurement sites. K-means cluster is used to group these time by space matrices into patterns of phase that are similar over trials. The traveling wave model is then fit to mean pattern indicated by each cluster. **a** The trial-averaged data from all the trials, showing a static pattern of activity (*vertical stripes*) that changes sign over the time cycle. **b** The sub-averaged data from each of the six clusters found by k-means. There is a wide variety of behaviors apparent, from traveling waves (B1) to standing waves (B6). The *top row* shows the raw MEG signal, in units of Tesla. The number of trials in each average is given by *n*. The *second row* shows the cosine of the trial-averaged phase. The *third row* shows the model wave estimated from the trial-averaged phase. *v* is the normalized velocity, *ξ* is the spatial frequency and *r* is the fit of the wave model to data. These example data are centered on 150 ms post-button press. The sensor ordering is shown by the *letters* on the *left* of *each graph*. ‘A’ is the most anterior site; ‘P’ is the most posterior, ‘L’ left, ‘R’ right and ‘S’ superior

Another example arises in the study of the receptive fields (RFs) of single neurons. Panel B,I shows the RF map for a neuron in the primary visual cortex, incorporating into the plot both *f*(*t*) and *g*(*x*). Here, *x* are the coordinates of visual space, but the same logic of space–time separability applies. Panels B,I,A and B,I,B show the time course of the neuron’s response, for two choices of *x*. The two curves differ only by scale, that is, the differing spatial location is evidenced by only a change in a weighting factor.

It is worth noting that, by definition, the domains of time (*t*) and space (*x*) of *f*(*t*) and *g*(*x*) do not change from moment to moment, nor over the time course of an experiment. Changes in activity patterns observed are understood as determined by changing weights (linear combinations) of the activation functions *f*(*t*) and *g*(*x*). More specifically, the practice in cognitive neuroscience is to make use of unchanging *f*(*t*) to analyze *g*(*x*), or unchanging *g*(*x*) to analyze *f*(*t*). When an effect in time is expected, this is to occur within a static spatial domain *x*, such that two conditions differ only in terms of a temporal variable. For example, an ERP peak at *x*_*A*_ takes longer to arise or reaches a higher value in one condition than another. Conversely, an effect on a spatial variable is expected to have the same time course across conditions of measurement. For example, a dipole is more prominent in one condition than in another; a BOLD signal is more widespread in one condition than in another.

We can now observe what is afforded by the assumption of space–time separability. If the temporal function does not change across experimental conditions, we can distinguish *A*_1_ from *A*_2_ … *A*_*n*_ in terms of only those components of the signal that are purely a function of space, i.e., *g*(*x*). This is what we do when we compare two snapshots of behavior, discounting their temporal courses, or two fMRI images. The converse case is where the spatial function does not change across conditions. In this case, the separability assumption enables us to characterize the typical temporal activation. Donders assumed that he needed only consider in his experiments the time course of the subject’s behavior, after holding everything else constant or considering it random fluctuation. In short, the difference method proves its usefulness by allowing the subtraction of only a subset of the signal components.

We also can now see the relationship between the difference method and trial-averaging. If *A*_1_ is held constant experimentally from trial to trial, then, in case other components in *A*_2_ … *A*_*n*_ vary, this can be disregarded as noise, which is removed by averaging. This step is justified if the variables we are averaging over are randomly distributed and uncorrelated with *A*_1_, which is a familiar assumption of statistical testing. As with the difference method, the assumption of space–time separability means we can undertake trial-averaging on the spatial or temporal component of the signal only. This may, for instance, be the time taken to press a button, which amounts to averaging over instances of *f*(*t*), assuming *g*(*x*) remains the same by virtue of our experimental procedures. Likewise, event-related potential (ERP) components are assumed to have a characteristic time course. Noise and other unwanted factors can be removed by averaging, leaving us with a topography, amplitude and latency characteristic of the ERP.

Conversely, cortical activation can be averaged over trials to produce an activation topography by holding *f*(*t*) constant or otherwise averaging over many *g*(*x*) instances. This averaged activation topography can be used in the EEG and MEG signals to estimate localized sources. The topography is assumed to reflect the relevant signal in *A*_1_, while the variation due to *A*_2_ … *A*_*n*_ is washed out by the trial-averaging. Similar reasoning applies to construction of activation maps in fMRI, except these are usually based on applying a combination of trial-averaging (within conditions) and the difference method (between conditions), keeping in mind that we first must have correlated the fMRI temporal signal with the model signal to extract each case of *g*(*x*).

It is worth emphasizing why the assumption of space–time separability is such a useful instrument. Reducing the dimensionality of the possible function space in which the data reside greatly reduces the complexity of the scientific problem. The problem now becomes simply a matter of how to transform the domain of either the time series, or the spatial topography, so that simple addition or subtraction can be applied in either of the functions. In general, the appropriate transformation of either domain can be parameterized by choosing a scaling factor (e.g., an effect is shorter or longer duration or has a larger spatial extent) and a translation (e.g., an effect occurs earlier or later in time or is displaced within the topography). We shall see this concretely in the examples to follow.

## Examples of the additive operations applied to space–time separable measurements

If we focus on time series, then, because time is one-dimensional, our activation function can be transformed by scaling and a time offset

$$\hat{f}(t) = f( \propto t + \beta )$$

Here, ∝ is the scaling factor and $\beta$ is the activation latency or temporal offset. Transformation of spatial functions is likewise usually limited to scaling and translation.

$$\hat{g}(x) = g(\zeta x + \eta )$$

In the case of fMRI, *ζ* describes the scaling of individual brains to fit a standardized coordinate system, such as Talairach coordinates, while *η* describes the exact position of a brain relative to the imaging device, having already been rotated into the correct orientation by virtue of putting the subject into the machine. Splitting the problem into temporal and spatial components, and considering only certain linear affine transformations of the domains, greatly simplifies the possible parameter space.

We will further illustrate these concepts with two examples from the EEG: the calculation of EEG power and ERP components. The measured data are denoted *d*. To obtain EEG power, we multiply the time series in *d* with a pair of sinusoids in quadrature and then integrate. We do this independently at each recording site, thereby throwing away relationships between the time series at different sites (other than the power). The sinusoids may be scaled allowing for quantification of a range of temporal frequencies.

In the case of ERPs, time *t* is discretely sampled and organized by experimental trials, *j*, and time within a given trial, *k*, to give *τ*_*jk*_. Again, we may consider each spatial location separately, given the assumption of space–time separability. We average over trials to isolate the ERP of interest

$$\hat{f}(\tau_{k} ) = \frac{{\sum\nolimits_{j = 1}^{n} {{\text{d}}[\alpha \tau_{jk} + \beta_{j} ]} }}{n}$$

Here *α* is (implicitly) assumed to be constant for each individual in a given task condition and therefore set to unity. The temporal offset for each trial, *β*_*j*_, is determined by the experimental setup, e.g., the inter-trial interval. The temporal function $\hat{f}$ is assumed to be revealed by averaging away other, more variable, components of the signal. If we wish, we may then find the difference in $\hat{f}$ between conditions (as in the case of mismatch negativity) or average over subjects. These further steps enable us to produce typical scalp maps of EEG power for a task or, having averaged over trials, we can submit the ERP data at multiple sites to a source localization procedure.

In short, additivity is what allows averaging and differencing of signals; space–time separability is what allows us to do so on the isolated temporal or spatial components of a signal. This assumption also plays a role in statistical analysis, when we assume that measurements can be treated as independent events. Condition 4 allows these events to be mapped into a common coordinate system. For spiking activity, local field potentials, EEG, MEG and fMRI, physical time and scalp locations or Talairach space are the objective coordinate system of our measurements. Condition 6, so obvious it is almost always left implicit, assures that these operations take place in domains that do not change. In statistics, samples are taken from the same population, not a population that is constantly changing. In the context of time series analysis, this is referred to the assumption of stationarity.

## Large-scale traveling waves in the cortex

Here we review evidence of traveling waves in brain activity; as we will observe, the evidence suggests that the additive assumption as usually applied in neuroscience does not hold. Traveling wave activity has been measured in the cortex at a number of scales, including columns (Livingstone 1996; Nauhaus et al. 2009), Brodmann areas (Benucci et al. 2007; Freeman and Barrie 2000; Gabriel and Eckhorn 2003; Rubino et al. 2006) and whole cortex (Alexander et al. 2006a, b; Ito et al. 2005; Manjarrez et al. 2007; Massimini et al. 2004; Nolte et al. 2008; Ribary et al. 1991). These waves have been found using a number of techniques, such as optical imaging (Benucci et al. 2007; Xu et al. 2007), LFP (Freeman and Barrie 2000; Gabriel and Eckhorn 2003; Livingstone 1996; Nauhaus et al. 2009; Rubino et al. 2006), EEG (Alexander et al. 2006a, b; Ito et al. 2005, 2007; Manjarrez et al. 2007; Nolte et al. 2008; Sauseng et al. 2002), MEG (Alexander et al. 2013; Burkitt et al. 2000; Ribary et al. 1991) and fMRI (Aquino et al. 2014; Bießmann et al. 2012; Lee et al. 2005).

We focus on traveling waves at the largest cortical scale, that is, 10–30 cm. Large-scale cortical waves have been shown to arise at a variety of frequencies, from the sub-delta through to gamma bands (Alexander et al. 2006a, b, 2013; Ito et al. 2005; Massimini et al. 2004; Ribary et al. 1991; Sauseng et al. 2002). These waves dominate cortical phase dynamics, that is, they explain more than 50 % of variance in phase. Previous work on large-scale patterns in EEG has shown that the phase vectors at specific frequencies form spatial patterns that are often of long wavelength (Alexander et al. 2006a, b; Ito et al. 2005, 2007); here long-wavelength means with a spatial period approximately equal to the scale of the measurement array: 10 to 30 cm. In a recent study (Alexander et al. 2013), the percentage of variance explained by the long-wavelength components was 62 % for MEG (first four eigenvectors), 69 % for EEG (first four eigenvectors) and 56 % for ECoG data (first three eigenvectors). Even though these large-scale waves thus correspond to the dominant eigenmodes of the phase data, their predominance in the signal is often overlooked. As we will explain, this is because it is washed out in the aforementioned common procedures involving averaging the data prior to or during analysis (Alexander et al. 2013).

An example traveling wave in MEG is shown in Video 1. The functional significance of TWs has been established by noting their close correspondence with the latency topography of known visual and auditory ERP components, such as the P1–N1 complex, P2–N2 complex, as well as the P3b (Alexander et al. 2006a, b, 2009; Anderer et al. 1996; Fellinger et al. 2012; Klimesch et al. 2007). For these event-related potential (ERP) components, the latency, temporal frequency and task dependency of evoked TW components are consistent with latency, temporal frequency and task dependency of the corresponding ERPs.

## The fly in the ointment

Traveling waves in the cortex have the form of patterns of phase that are ordered in space and time.

We will illustrate this point by considering the latency of the peak of a wave. The peak of the wave moves forward in time. That is, if we wait at a certain point where the peak has not arrived yet, the peak will arrive and then pass. The peak of the wave also moves across space. If we start at the location where the peak currently resides, and follow the peak, we will move with the wave across space. Hence, the wave is ordered in both space and time.

For convenience, we can picture this shape of the wave as a cosine. It is possible then to describe the shape of the wave as either

$$\hat{f}(t) = {\text{cos(}}\alpha t + \beta ) ,$$

at a given location, or

$$\hat{g}(x) = {\text{cos(}}\zeta x + \eta ) ,$$

at a given time.

Here, *x* can be thought of as measuring distance along a straight line, pointing in a particular direction. *α* and *ζ* simply scale the wave in time and space, corresponding to the temporal and spatial frequencies, respectively.

Although we now have expressions for $\hat{f}$ and $\hat{g}$, these equations do not capture the relationship between the ordering of the wave in space and time. This behavior can be captured by

$$\hat{A}(t,x) = \cos (\alpha t + \zeta x + \gamma )$$

where *α* and *ζ* parameterize the symmetry of the wave behavior in space and time and *γ* = *η* − *αt*_0_ = *β* − *ζx*_0_. This equation implies that a shear transformation of the coordinate system is necessary to make the function separable. For example, the velocity of the wave is the ratio of *α* and *ζ*, i.e., the slope of the shear. The wave cannot be described if we limit ourselves to scaling and translations of the temporal and spatial domains. In other words, the form of the wave is not space–time separable.

Crucially, the exact direction of the coordinate transformation changes from moment to moment, as the direction of the wave changes. The underlying parameters, specifying transformations of the domain, are not constant.

**Fig. 3:**
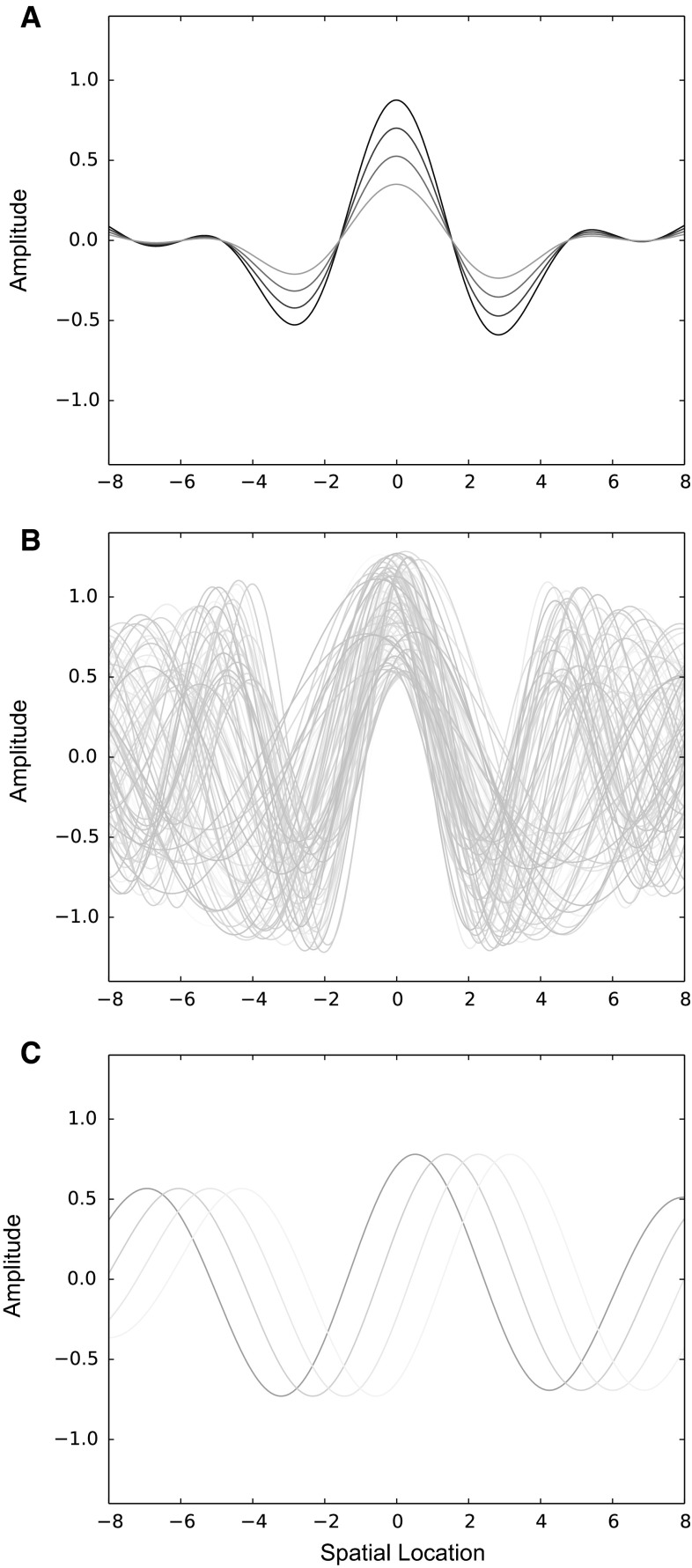
Schematic illustration of components of the EEG signal. Space is represented on the *horizontal axis*, signal amplitude on the *vertical axis*. The signal at different times is represented by *multiple curves*. **a** Time-locked and space-locked components are enhanced when signal is averaged over trials. The trial average at sample time *t* = 1 is shown in darkest gray, with a maximum amplitude over *x* = 0. Over consecutive samples (*lighter gray*), the trial averages retain the same spatial envelope, and the amplitude varies. **b** Individual trials from which (**a**) is computed at time *t* = 1. All these curves are averaged together to form one curve in (**a**). The amplitude of the signal declines from the spatial location of the maximum, but less steeply than appears in (**a**). The *curves* have more jitter near the edges of the array, while they are more phase-locked in the center. Most of the amplitude information in (**a**) is due to the degree of phase jitter across trials, rather than changes in amplitude, per se. **c** An individual trial from the set shown in (**b**), over times *t* = 1 to 4. The sequence across consecutive time samples is indicated by lightening of the *gray curve*. While the amplitude of the signal declines slowly from the center, the most prominent component of the signal is the motion of the wave from *left* to *right*. The degree of jitter in (**b**) can be understood as due to different velocities of wave propagation in different trials. The maximum amplitude in (**a**) reflects the coordinate in space and time at which the peak in the waves most consistently intersect each other. The trial averages in (**a**) are therefore entirely consistent with the time course of the traveling wave, shown in this example, when averaged over the many trials shown in (**b**). However, (**a**) and (**c**) are quite different spatiotemporal patterns. In fact, the amplitude of (**a**) is a measure of the degree of constructive/destructive interference of the kind of signal shown in (**c**)

The situation becomes more complicated if we wish to measure several different kinds of dynamical regimes. For example, it is possible to capture the behavior of both standing waves and traveling waves. Here, the equation becomes

$$\hat{A}(t,x) = p { \cos }(\alpha t + \zeta x + \gamma_{p} ) + m { \cos }(\alpha t - \zeta x + \gamma_{m} )$$

where *p* and *m* are weightings given to the traveling wave and its space-reversed twin, respectively, and *η*_*p*_ and *η*_*m*_ are their respective spatial offsets. Examples of waves described by this equation are given in Fig. 3. Similar complexities arise in the characterization of spiral waves in the cortex (Ito et al. 2005; Prechtl et al. 2000).

When trial averages of EEG signal are made, thereby invoking the assumption of space–time separability, this enhances time-locked and space-locked portions of the signal (Alexander et al. 2013), as illustrated in Fig. 3 (upper). The individual trial signals, prior to being composed into the trial average, show a high degree of ‘noise,’ as illustrated in Fig. 3 (middle). It is often assumed that high variation in phase represents portions of the signal that are not relevant to the experimental task (Arieli et al. 1996; Ray and Maunsell 2011). However, when understood as snapshots of traveling waves, the jitter in phase represents changing patterns of velocity from trial to trial, which may or may not be related to the experimental task (Fig. 3, lower). If time and space coordinates are taken as the appropriate domains to average over, then much of the traveling wave signal is lost due to averaging over changes in the dynamics of the wave, i.e., by lumping different cases of *α* and *ζ* together.

Since the axis of symmetry of the wave changes from moment to moment, the correct coordinate transformations cannot be assumed but must be calculated from the available data. Recent research supported the finding that properties of the traveling waves change from trial to trial, depending on task demands. Waves can differ by frequency, wavelength, onset latency and velocity (Alexander et al. 2006a, b, 2009, 2013). The direction of the wave, in particular, is task dependent. Examples of this task dependency are illustrated by the traveling waves associated with the P2/N2 and P3b event-related potentials (Alexander et al. 2006a, b). The P3b, for example, has latency in adult subjects that increases in an anterior-to-posterior direction across the scalp. This is due to delta-band traveling waves at the individual trial level, which have as their predominant mode an anterior-to-posterior direction (Alexander et al. 2006a, b).

These task-related events, however, do not fully constrain the direction of the wave. For example, the traveling waves associated with the P3b consist of two modes: one anterior to posterior, and one posterior to anterior (Alexander et al. 2009). The pattern of P3b latencies across the scalp is determined by which mode is dominant, and the dominant mode varies with age, among other factors. Consistent with the accumulated data on ERPs, the waves reproduce the peak and location of time characteristic of the event-related potential via constructive interference across trials at this time and location. But the direction and other properties of the wave can (and do) otherwise vary. This relationship between ERPs and traveling waves is illustrated in Video 2.

Here we may now point to one concrete finding where TWs provide additional insight beyond that provided by ERPs. While the P3b is generally considered to be generated by static, localized regions of activity in the temporal and parietal cortex (Polich 2007), it has long been observed that the P3b has a latency gradient across the head and that this latency gradient changes with age (Anderer et al. 1996). This effect has been explained as the relative strength of the two TW modes. In children and adolescents, the posterior-to-anterior direction is more dominant, while the reverse is true in older adults (Alexander et al. 2006a, b, 2008, 2009). This developmental trajectory has intriguing implications in terms of white matter maturation in the frontal cortex and may also be reflected in disorders of development. Specifically, a preponderance of posterior-to-anterior P3b-related TWs has been implicated in symptoms of psychomotor poverty in first episode schizophrenia (Alexander et al. 2009).

The variability in direction of TWs means that measurement coordinates must first be transformed in order for quantities to obey the additive assumption. This situation is analogous to the finding that simple cells in the primary visual cortex are not space–time separable. The traveling wave functions appear as rotated or sheared versions of functions that are space–time separable. In order to average over different direction-selective cells (or traveling waves) to produce the typical activity function, the coordinate systems of each cell (or wave) needs to be first aligned.

We may describe this alignment in general terms by the transformation *Q*

$$\left[ {\begin{array}{*{20}c} u \\ v \\ \end{array} } \right] = Q\left[ {\begin{array}{*{20}c} t \\ x \\ \end{array} } \right]\quad Q:{\mathbb{R}}^{4} \to {\mathbb{R}}^{4}$$

where *Q* is a transformation of the domain of the four-dimensional spatiotemporal coordinate system. In the case of traveling waves, *Q* includes a shear, as well as a temporal offset and scaling. After this transformation, we may rewrite our activation functions, thus:

$$\hat{A}(t,x) = \tilde{A}\left( {Q(t,x)} \right) = \tilde{A}\left( {u,v} \right) = \tilde{f}\left( u \right) \times \tilde{g}(v)$$

where $\tilde{f}$ and $\tilde{g}$ are linearly independent and can be summed and subtracted. A concrete illustration of the class of transformations of the coordinate system, *Q*, can be illustrated by tilting one’s head by 20° to the left while viewing Fig. 1,C,II. Now, the useful properties and simplifications previously imbued by space–time separability again become available.

The procedure for detecting traveling waves is one where the data determine *Q*, and *Q* varies from moment to moment. *u* and *v* can be thought of as a coordinate system determined by the ongoing dynamics of the cortical system rather than that determined solely by the position of the cortical system in space and time.

We may summarize this argument by noting that the purpose of experimentation is to hold extraneous factors constant (Bhaskar 1997). Evidence from the study of cortical traveling waves indicates that characterizing the temporal or spatial components of the signal separately is not sufficient to hold the relevant factors constant. It is not simply a matter of dealing with unwanted, uncontrolled factors, which for practical considerations can be ignored, such as air resistance of objects in freefall. Since traveling waves account for a large proportion of the variance in measured phase, and since traveling waves are found at many spatial and temporal scales of cortical organization, we suggest that cortical activity cannot be satisfactorily understood using the assumption of space–time separability and associated logic described by (1)–(6). In other words, the relevant signal is not space–time separable, and therefore, assuming space–time separability destroys relevant signal (Alexander et al. 2013). As we stated at the outset, our working assumption is that space–time separability is akin in status to the four humors, that is, it reflects a preparadigmatic view, rather than being a paradigm in need of revolution (Kuhn 1970). Like others (Kampis 1991; Maturana and Varela 1980; Skarda and Freeman 1987), we question the very notion that neurological entities are events occur at certain locations and times, rather than being comprised of trajectories that extend over locations and times. These issues can only be decided as scientific discovery unfolds, when new approaches produce powerful results that are not explicable within the previous framework.

## Conclusion

We suggest that cortical activity in general is not space–time separable. The precise transformation to the coordinate system that would allow space–time separability changes from moment to moment. These observations regarding cortical activity have a number of important implications.

Trial averages (as they are usually taken in neuroscience) do not just reduce noise, but also wash out task-related activity that due to the changing coordinate system is non-additive. This can be seen in Fig. 2, where the shape of the trial-averaged wave does not look like any of the waves found by clustering together similar pattern of phase. Subtraction of activation topographies between conditions distinguishes only some of the signal; some true differences are missed in the ‘noise’ of the changing coordinate system. This point can be illustrated by considering the space–time inseparable RFs of direction-selective neurons. As described previously, the difference between the two curves in 1,C,I A and B is a scaling factor. The factor can be found by dividing pairs of values in the two curves (*f*_A_/*f*_B_ = −1). In the case of Fig. 1,B,II A and B, the difference between the two curves turns out to be not simply a scaling factor; due to the absence of space–time separability, the two curves are rather different in shape. In order to retrieve the correct scaling factor, we would first have to rotate the underlying coordinate system according to *Q* and only then would the two curves differ by a constant weighting factor. In other words, it is the transformation of the coordinate system by *Q* takes us from space-time inseparability to a separable system. While this is easily illustrated in the case of direction-tuned RFs, the evidence from traveling waves in the cortex is that the same applies to large-scale cortical signals.

Many of the available techniques in neuroscience are mathematically sophisticated; this also means that the assumptions of the techniques are explicitly laid out. For example, most fMRI studies make use of a convolution model based on the hemodynamic response function, explicitly stating as their core assumption space–time separability within an unchanging coordinate system for the hemodynamic BOLD response. A number of studies are suggesting that this might not be justified (Aquino et al. 2014; Bießmann et al. 2012). The assumption of space–time separability applies to independent component analysis (De Lathauwer et al. 2000). However, according to the argument laid out here, its use in analysis of EEG signals (Delorme and Makeig 2004) of cortical origin is problematic, since the independent components do not capture the non-space–time separable character of the cortical signal. Likewise, coherence measures in EEG and MEG assume that the signal is that part of the measurement which is consistent across time-locked events, for a given site or pair of sites. If so, the measurements already reside in a coordinate system such that the signal is revealed by application of such cross-trial measures. But if not, their application may provide misleading results. The prominence of traveling wave activity suggests that this might be the case.

As we have illustrated with the case of direction-selective RFs, once the proper coordinate system has been defined, and the transformation *Q* applied, then the additive assumption may be safely applied. Some progress has been made toward the goal of analyzing wave activity differences across defined brain states, such as rest (Ito et al. 2005, 2007), deep sleep (Massimini et al. 2004) and working memory (Fellinger et al. 2012; Sauseng et al. 2002). Single-trial TWs have also been used to uncover genetic differences in brain activity (Alexander et al. 2007), differences across age groups and clinical groups and in correlations with clinical symptoms (Alexander et al. 2006a, b, 2008, 2009). Of course we may still be wrong in the choice of *Q*, but this is still better than assuming that *Q* is the identity matrix, i.e., the object of study is already in the correct coordinate system.

If cortical activity is not space–time separable, then it seems likely that neither are perception or action. We take the view that measurements in neuroscience are not of events but of trajectories (McKenna et al. 1994; Skarda and Freeman 1987). This insight defers the analysis of typical behavior and differences in behavior until the trajectory space is defined. If we consider something as simple as a button press in a reaction time experiment, it cannot be assumed that the relevant coordinates for that event are the spatial location of the button, the time at which the press occurred and the latency and location of the associated cortical activity. Our arguments suggest that the total behavioral trajectory must be measured, along with the cortical trajectory, and the underlying coordinate system determined from these data. Similar conclusions have been drawn from analysis of reaction time data across multiple experiments (Van Orden et al. 2003). Behavior is not separable into independent components, but is correlated across multiple timescales (Van Orden and Holden 2002), similarly for cognition and perception. Cortical traveling waves provide a natural mechanism for this process at shorter timescales, linking instantaneous neural activity to wave cycles extending over 100 s of milliseconds, and linking localized cortical function to activity over the whole cortex.

Our results therefore bring into question Donders’ subtraction method, which is the basis for much of neuroscience and psychology experimentation. According to our analysis, Donders’ case of entering the room by different doors is the rule rather than the exception. Perhaps Donders was right to be suspicious when he noted the large variability in experimental measurements, something which bedevils psychological experimentation to this day. Future unraveling of these issues will be determined by the amount of signal explained by the different approaches, along with that signals’ explanatory power in behavior across settings, tasks, developmental stages, clinical groups and genetic diversity.

## Electronic supplementary material

Supplementary material 1 video 1 single-trial traveling wave in the MEG. The video shows the left view of the sensor array on the left side of the image and the right view of the sensor array on the right side of the image. The data were band-pass filtered with center frequency 9.2 Hz, prior to amplitude normalization and taking the cosine of the phase. The video shows a traveling wave that originates on the inferior left of the array and travels to the lower right anterior of the array. Color scale is cosine of phase (black = −1, white = +1) (MPG 1190 kb)

Supplementary material 2 video 2 Illustration of how the averaging of TW components over trials can lead to typical ER-type activation patterns. The four animations in the top left (numbered 1-4) show TW components as the cosine of the phase (hot to cold colors). The four TWs have four different trajectories, as indicated underneath each plot. In the four TWs, the peaks are not localized in space or time, but move as a coherent band over time. The other five colored animations show the effects of averaging the waves in various combinations. The average of the four waves has a peak in magnitude that is localized both in space and in time (‘Waves mean’ plot). Various other behaviors are possible in the trial averages, depending on the weights given to the different TW components (e.g., ‘Waves 1+2’; ‘Waves 3+4’). The rightmost column shows measurements taken from individual recording sites. In general, they are unable to distinguish between the different dynamical patterns seen in animations 1 to 4. The exceptional time series is the measurement site with zero magnitude in activity (‘+’ symbols) in the bottom-right plot. However, this behavior does not exist in the component waves, 1 to 4 (MPG 4160 kb)
